# Soft Corals-derived Dihydrosinularin Attenuates Neuronal Apoptosis in 6-hydroxydopamine-induced Cell Model of Parkinson's Disease by Regulating the PI3K Pathway

**DOI:** 10.2174/0109298673323198240823070219

**Published:** 2024-09-03

**Authors:** Zhi-Hong Wen, Ya-Jen Chiu, San-Nan Yang, Tzu-Yi Huang, Chien-Wei Feng, Nan-Fu Chen, Chun-Sung Sung, Wu-Fu Chen

**Affiliations:** 1 Department of Marine Biotechnology and Resources, National Sun Yat-Sen University, Kaohsiung, 804201, Taiwan;; 2 Department of Neurosurgery, Kaohsiung Chang Gung Memorial Hospital and Chang Gung University College of Medicine, Kaohsiung, 833301, Taiwan;; 3 Department of Pediatrics, E-DA Hospital, School of Medicine, College of Medicine I-Shou University, Kaohsiung, 82445, Taiwan;; 4 Department of Obstetrics and Gynecology, Kaohsiung Medical University Hospital, Kaohsiung Medical University, Kaohsiung, 80756, Taiwan;; 5 Department of Surgery, Division of Neurosurgery, Kaohsiung Armed Forces General Hospital, Kaohsiung, 80284, Taiwan;; 6 Department of Anesthesiology, Division of Pain Management, Taipei Veterans General Hospital, Taipei, 112201, Taiwan

**Keywords:** Dihydrosinularin, parkinson's disease, 6-hydroxydopamine, PI3K signaling pathway, neuroprotection, natural bioactive molecule

## Abstract

**Background:**

Parkinson's disease (PD) is an irreversible, progressive disorder that profoundly impacts both motor and non-motor functions, thereby significantly diminishing the individual’s quality of life. Dihydrosinularin (DHS), a natural bioactive molecule derived from soft corals, exhibits low cytotoxicity and anti-inflammatory properties. However, the therapeutic effects of DHS on neurotoxins and PD are currently unknown.

**Objective:**

This study investigated whether DHS could mitigate 6-hydroxydopamine (6-OHDA)-induced neurotoxicity and explored the role of neuroprotective PI3K downstream signaling pathways, including that of AKT, ERK, JNK, BCL2, and NFκB, in DHS-mediated neuroprotection.

**Methods:**

We treated the human neuroblastoma cell line, SH-SY5Y, with the neurotoxin 6-OHDA to establish a cellular model of PD. Meanwhile, we assessed the anti-apoptotic and neuroprotective properties of DHS through cell viability, apoptosis, and immunostaining assays. Furthermore, we utilized the PI3K inhibitor LY294002 to validate the therapeutic target of DHS.

**Results:**

Based on the physicochemical properties of DHS, it can be inferred that it has promising oral bioavailability and permeability across the blood-brain barrier (BBB). It was demonstrated that DHS upregulates phosphorylated AKT and ERK while downregulating phosphorylated JNK. Consequently, this enhances the expression of BCL2, which exerts a protective effect on neuronal cells by inhibiting caspase activity and preventing cell apoptosis. The inhibition of PI3K significantly reduced the relative protective activity of DHS in 6-OHDA-induced neurotoxicity, suggesting that the neuroprotective effects of DHS are mediated through the activation of PI3K signaling.

**Conclusion:**

By investigating the mechanisms involved in 6-OHDA-induced neurotoxicity, we provided evidence concerning the therapeutic potential of DHS in neuroprotection. Further research into DHS and its mechanisms of action holds promise for developing novel therapeutic strategies for PD.

## 1 INTRODUCTION

Parkinson's disease (PD) is the second most prevalent neurodegenerative disease after Alzheimer's disease (AD) and is a major source of severe medical, social, and financial burdens [1, 2]. There is currently no effective therapy for PD. Clinically, only symptomatic treatments are available, but their long-term application typically results in substantial adverse effects [3]. Thus, one of the most pressing challenges is the active development of novel neuroprotective therapies for PD.

Pathologically, PD is characterized by the presence of Lewy bodies and Lewy neuritis containing α-synuclein in subcortical brain regions [4]. Although the molecular mechanisms causing dopamine neuron degeneration that leads to PD are unknown, many research reports indicate that oxidative stress, mitochondria dysfunction, and the inflammatory process all play important roles in inducing dopamine neuron apoptosis in the substantia nigra, contributing to PD progression [5]. 6-Hydroxydopamine (6-OHDA) is a hydroxylated analog of the neurotransmitter dopamine, which is toxic to the mitochondrial complex I [6]. It also induces the production of superoxide and free radicals, causing oxidative stress and chromosomal damage or mutations [7, 8]. Because of the similarities between the effects of 6-OHDA and the neuronal death pathway in PD, it is frequently employed to create *in vitro* and *in vivo* PD models [9, 10]. Previously, we used this cellular model to study the neuroprotective effects of sponge-derived stellettin B (SB) and soft coral-derived 11-dehydrosinulariolide (11-de) as treatments for PD [10, 11].

Phosphoinositide 3-kinase (PI3K)/protein kinase B (AKT) and extracellular signal-related kinase (ERK) signaling not only increases cell viability by phosphorylating cAMP response element-binding protein (CREB) but also prevents apoptosis by deactivating the Bcl-2 agonist of cell death (BAD) and caspase 3 in neuronal cells [12-14]. The PI3K/AKT signaling pathway can deactivate c-Jun N-terminal kinase (JNK), leading to the upregulation of BCL2 expression and downregulation of BAD activation, ultimately contributing to cell survival [15, 16]. Furthermore, the suppression of PI3K results suppresses the ERK signaling pathway, thus impacting neuronal survival [17]. Given these considerations, activating PI3K/AKT, ERK, and JNK signaling to decrease neuronal death can be considered a therapeutic strategy for PD.

Marine-derived compounds have distinct structural and chemical properties not found in terrestrial natural products [18]. Despite the plethora of marine compounds, few have been investigated for clinical applications in neurodegenerative diseases [19]. Dihydrosinularin (DHS) is a cembrene diterpenoid compound and was first isolated from the soft coral *Sinularia querciformis* in Australia in 1977 [20]. It presents few cytotoxic properties and can be obtained from the wild and cultured type of the finger-soft coral *S. querciformis* [21, 22]. Previous studies indicated that the IC_50_ of DHS in breast (MDA-MB-231), lung (H1299), liver (Ha22T), and six oral cancer cell lines was more than 200 μM [23, 24]. In a previous *in vitro* inflammatory model, DHS significantly reduced the production of inflammatory mediators in lipopolysaccharide (LPS)-stimulated macrophages [22]. Since several studies have established the substantial impact of inflammatory responses in PD and as one of the primary factors contributing to neuronal death [25], we hypothesized that DHS, characterized by its anti-inflammatory properties and low cytotoxicity, could potentially offer neuroprotective advantages. The primary goal of this study was to investigate the neuroprotective effects and fundamental processes underlying the antineuronal apoptotic properties of DHS. We subsequently investigated whether DHS had neuroprotective effects and influenced apoptosis and PI3K/AKT, ERK, and JNK signaling pathways in the 6-OHDA-induced neurotoxicity SH-SY5Y model.

## 2 MATERIALS AND METHODS

### 2.1 Dihydrosinularin and Chemicals

The method of dihydrosinularin (DHS) extraction was modified from that of Chen *et al.* [22]. Soft coral *Sinularia querciformis* specimens were initially collected off the coast of Pingtung, southern Taiwan. The soft coral (wet weight = 1.3 kg) was sliced and treated with ethanol (EtOH) at room temperature to produce a crude extract weighing 35.6 g, which was washed first with *n*-hexane (19.4 g) followed by ethyl acetate (EtOAc, 2.1 g). The EtOAc phase was applied to silica gel column chromatography (Si CC). Elution was carried out with a gradient solvent system containing *n*-hexane, followed by increasing the polarity mixtures of *n*-hexane and EtOAc, pure acetone, and pure methanol for using them as eluting solvents. The process yielded fractions. The fraction 14 was separated by Si CC and eluted with an isocratic solvent system, *n*-hexane/EtOAc mixture (4:1). The process yielded eight fractions. Fractions 14D and 14E were combined and purified by normal phase high-performance liquid chromatography (NP-HPLC) with an isocratic solvent system, *n*-hexane/ acetone mixture (4:1; flow rate = 3 mL/min) to afford DHS (Supplementary Fig. **S1**). 6-hydroxydopamine (6-OHDA, dissolved in 2% l-ascorbic acid) and LY294002 (PI3K inhibitor) were supplied by Sigma-Aldrich Co. (St. Louis, MO, USA).

### 2.2 Physicochemical Property Computation

The molecular weight (MW), hydrogen bond donor (HBD), hydrogen bond acceptor (HBA), predicted octanol-water partition coefficient (cLogP), and polar surface area (PSA) of the DHS were determined using the online software ChemDraw (Available at: http://www. perkinelmer.com/tw/category/chemdraw).

### 2.3 Cells and Culture

Human neuroblastoma SH-SY5Y cells (American Type Culture Collection; No. CRL-2266) were maintained in Dulbecco’s modified Eagle’s medium/nutrient mixture F12 (DMEM/F12) supplemented with 10% fetal bovine serum (FBS) (Thermo Fisher Scientific), with 20 U/mL penicillin-streptomycin added to the growth medium. Cells were cultured in a CO_2_ incubator (Thermo Electron Corporation, Waltham, USA) at 37°C with 5% CO_2_.

### 2.4 Alamar blue Assay for Cell Viability and Cell Protection Detection

The following experiments were performed based on previously established methods [11]. SH-SY5Y cells were seeded at a density of 2 × 10^4^ cells/well in 96-well plates. The treatment was initiated after 24 h of incubation at 37°C, 5% CO_2_, and 95% air in a CO_2_ incubator when the cells had completely adhered to the bottom of the culture plate. The cells were subsequently treated with 6-OHDA for 15 h, after which 10% alamarBlue (Invitrogen, Waltham, MA, USA) was added and incubated for another 3 h. To assess the toxicity of 6-OHDA on neuronal cells, the fluorescence intensity was evaluated using a fluorescent microplate reader at 560/590 nm (excitation/emission) (BMG Labtech, Ortenberg, Germany). To determine whether marine natural products exert a protective effect on neuronal cells, the cells in the treatment group were pretreated for 1 h before incubation with 6-OHDA. The relative protection as a percent was calculated using the following formula: 100×[(optical density (OD) of 6-OHDA/DHS-treated cells−OD of 6- OHDA-treated cells) / (OD of control cells−OD of 6- OHDA-treated cells)] [26].

### 2.5 Analysis of Cell Apoptosis

Assessment of cell apoptosis was performed using the Hoechst stain from the CaspaTag™ Caspase 3, 7 *In Situ* Assay Kit (Sigma, St. Louis MO, USA), following a previously reported cell treatment process. The cell culture medium was supplemented with 0.5 µl of Hoechst dye (0.5% *v/v*) Hoechst dye, and incubated for 5 min. Following this, the cell culture medium was removed, and the cells underwent two rounds of washing using 1x Wash buffer. Subsequently, a diluted fixative was added to fix the cells. The apoptotic ratio of each set of cells was measured using the method published by Zhao *et al.* in 2007 [27]. Five fluorescent images and five corresponding bright-field images were captured for each well using an inverted fluorescence microscope (Leica, DMI 3000 B), with cells taken randomly at the same spots. After counting the cells in each image, the ratio of apoptotic cells was determined by dividing the number of cells showing a positive signal for apoptosis by the total number of cells.

The activity of caspases 3 and 7 was evaluated in separate cell groups after a 1 h pretreatment with the tested compounds or dimethyl sulfoxide (DMSO, as a control). Next, 6-OHDA (20 µM) was added to induce neurotoxicity. Depending on the experiment, the cells were incubated in a CO_2_ incubator for 0, 3, 6, 8, 12, and 18 h. Cell apoptosis was assessed using the Caspase 3, 7 *in Situ* Assay Kit. The methods used for imaging and analysis have been described previously.

### 2.6 Effects of PI3K Inhibitor LY294002 on DHS-induced Neuroprotection

On the first experiment day, SH-SY5Y cells were seeded in a 96-well plate with a density of 2 × 10^4^ cells per well. On the second day, the PI3K inhibitor, LY294002 (10 µM) and 6-OHDA (20 µM), were sequentially added as described after incubation for 1 h. Cell viability analysis was conducted on day 3 by staining the cells with alamar blue according to the indicated procedure.

### 2.7 Western Blot Analysis

A lysis solution comprising 50 mM Tris-HCl pH7.5, 150 mM NaCl, 2% Triton X-100, 100 μg/mL phenylmethylsulfonyl fluoride, and 1 μg/mL aprotinin was utilized to extract total proteins from SH-SY5Y cells. Protein samples were quantified using a DC™ Protein Assay Kit (Bio-Rad, Hercules, CA, USA) and then separated on 10% SDS-PAGE. Proteins were then transferred onto polyvinylidene difluoride (PVDF) membranes (Millipore, Billerica, MA, USA) using reverse electrophoresis. After blocking with 5% skim milk solution dissolved in TTBS buffer (Tris-Tween buffer saline; 20 mM Tris, 137 mM NaCl, 0.1% Tween 20, pH 7.4), the membrane was probed with primary antibodies against p-ERK (1:1500; Cell Signaling Technology, USA), ERK (1:500; Cell Signaling Technology), p-AKT (1:1000; Cell Signaling Technology), AKT (1:1000; Abcam, Cambridge, CB, UK), BCL2 (1:1500; BD Biosciences, USA), β-actin (1:1000; Sigma, USA), or NF-κB (1:1000; LabFrontier, Korea). The immunocomplexes were subsequently detected by adding a chemiluminescent substrate (Millipore, USA). Protein bands were captured and analyzed using an imaging analysis system (UVP, BioSpectrum AC Imaging System, USA and Canada).

### 2.8 Immunocytochemistry for Analysis of NF-κB Translocation

As described, on day 1, SH-SY5Y cells were seeded onto a 24 mm x 24 mm coverslip (Assistant, Germany); subsequently, on day 2, cells were treated with 10 µM DHS, and neurotoxicity was induced with 20 μM 6-OHDA [11]. On day 3, cells were fixed in 4% paraformaldehyde for 5 min on ice, permeabilized in TTBS buffer for 5 min, and blocked in blocking buffer for 40 min (Vector Laboratories, VECTASTAIN ABC kit). Cells were then stained with NFκB primary antibody at room temperature for 2 h, followed by secondary antibodies specific to the target protein at room temperature for 30 min. After staining the nuclei with 4’-6-diamidino-2-phenylindole (DAPI) for 15 s, samples were dried using a fume hood and then sealed with a mounting medium. The completed slides were placed under a Fully Automated Upright Microscope System (Leica, DM6000 B) to examine fluorescence, and the results were recorded using a SPOT RT Slider Scientific Digital Camera System (Diagnostic, RT Slider SPOT, USA). Each sample was imaged in a randomized manner, ensuring consistent positioning to allow the capturing of a series of five distinct fluorescent signals. After analyzing the cell count in each image, the ratio of NF-κB translocation was calculated by dividing the number of cells showing nuclear translocation of NF-κB by the total cell number. Finally, the average ratio of cells showing NF-κB nuclear translocation for each well was calculated, as reported by Levites *et al.* in 2004 [28].

### 2.9 Statistical Analysis

Data are presented as the mean ± the standard deviation of three independent experiments. Group differences were assessed using a two-tailed Student's *t*-test or one-way ANOVA with a post-hoc Tukey test, as necessary. Statistical significance was set at *p*-values less than 0.05.

## 3 RESULTS

### 3.1 Bioavailability and Cytotoxicity of DHS

The Lipinski rule-of-five, which includes a molecular weight (MW) of ≤500 Da, ≤5 hydrogen bond donors (HBD), ≤10 hydrogen bond acceptors (HBA), and a calculated LogP (octanol-water partition coefficient) of ≤5, is widely utilized to predict the oral bioavailability of medications [29]. Additionally, a topological polar surface area (tPSA) of less than 150 Å^2^ is necessary for molecules to permeate the blood-brain barrier (BBB) and exert effects in the central nervous system (CNS) [30]. The structures, formulas, and molecular weight of DHS are illustrated in Fig. (**1A)**. DHS adheres to Lipinski’s criteria for drug-like compounds. Furthermore, since the PSA is 59.06 Å^2^, DHS is expected to penetrate the BBB.

**Fig. (1):**
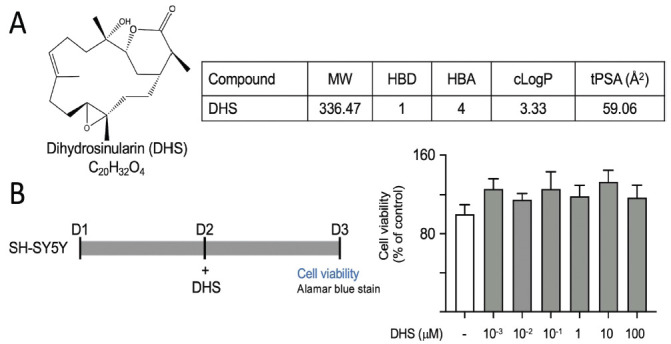
Properties of dihydrosinularin (DHS). (**A**) Structure, formula, molecular weight (MW), hydrogen bond donor (HBD), hydrogen bond acceptor (HBA), calculated octanol-water partition coefficient (cLogP), and topological polar surface area (tPSA) of DHS. (**B**) DHS cytotoxicity against SH-SY5Y cells using alamar blue assay. Cells treated with DHS (10^-^^3^-100 µM) for 18 h. For normalization, the relative viability of untreated cells is presented as 100%.

In addition, to gain insights into the impact of DHS on SH-SY5Y cells, we employed the alamar blue assay to evaluate the potential toxicity of different concentrations of DHS (Fig. **1B**). The cell viability of DHS (10^-3^-100 µM)-treated SH-SY5Y cells ranged from 114% to 133%, indicating a low level of cytotoxicity. Notably, there was a small increase in cell viability with DHS treatment, although this increase was not statistically significant compared to the control group. This finding suggests that DHS has the potential to support cell growth.

### 3.2 Protective Effects of DHS in 6-OHDA-induced Neurotoxicity

**Fig. (2):**
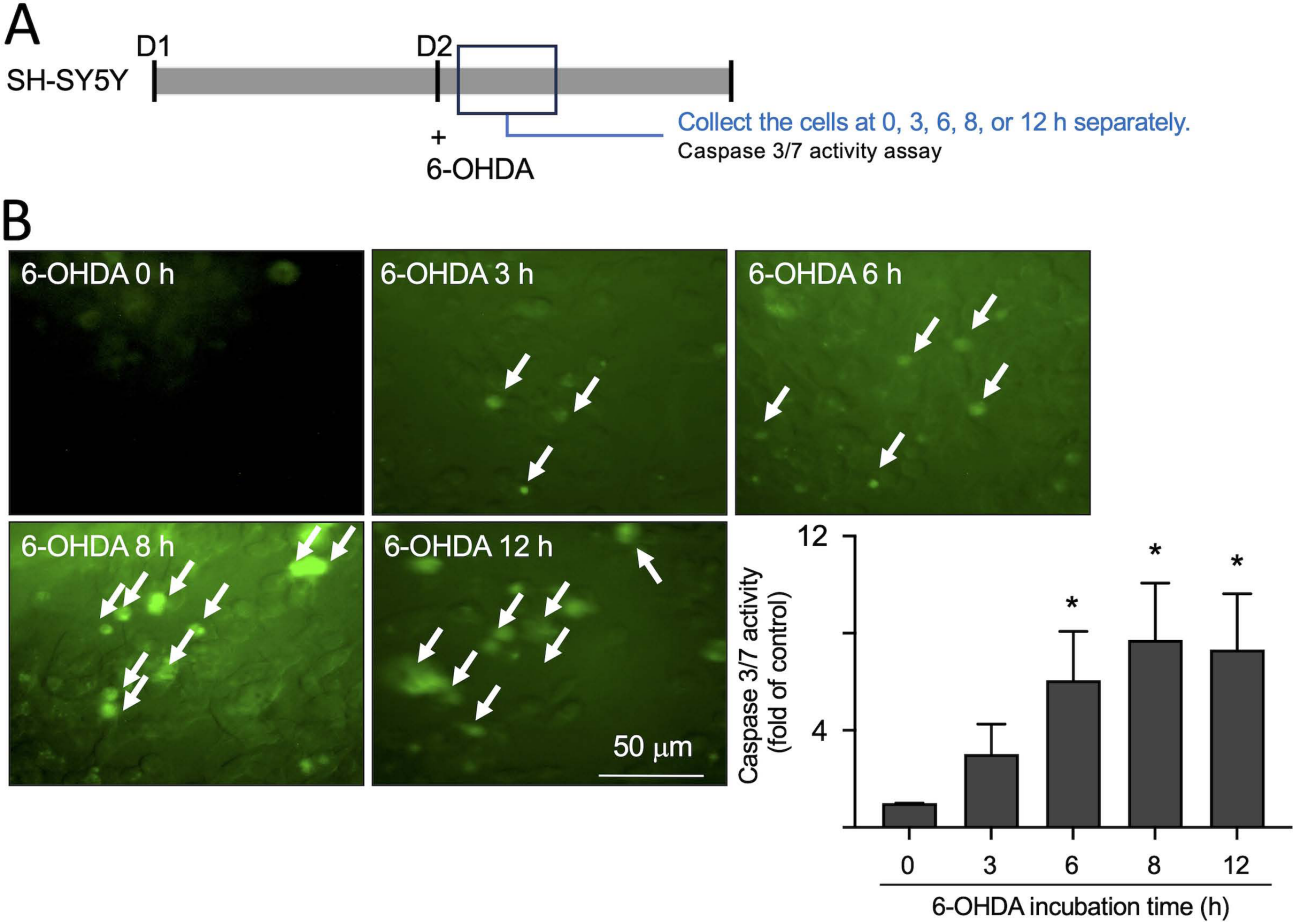
The impact of 6-OHDA on caspase 3 and caspase 7 activation in SH-SY5Y cells at various time points. (**A**) Experimental flow chart. On day 1, cells were plated. On day 2, after adding 20 µM of 6- hydroxydopamine (OHDA) to the cells, caspase 3/7 activity (CaspaTag™ Caspase 3, 7 *in situ* assay kit) was assessed at 0, 3, 6, 8, and 12 h. (**B**) Microscopic images and quantitative analysis of 6-OHDA (20 µM) effect on caspase 3/7 activation in SH-SY5Y cells at different time points. Activated caspase 3/7 was visualized using fluorescence microscopy with a green fluorescence signal, indicated by white arrows. **Note:** *p < 0.05 compared with the untreated group.

**Fig. (3):**
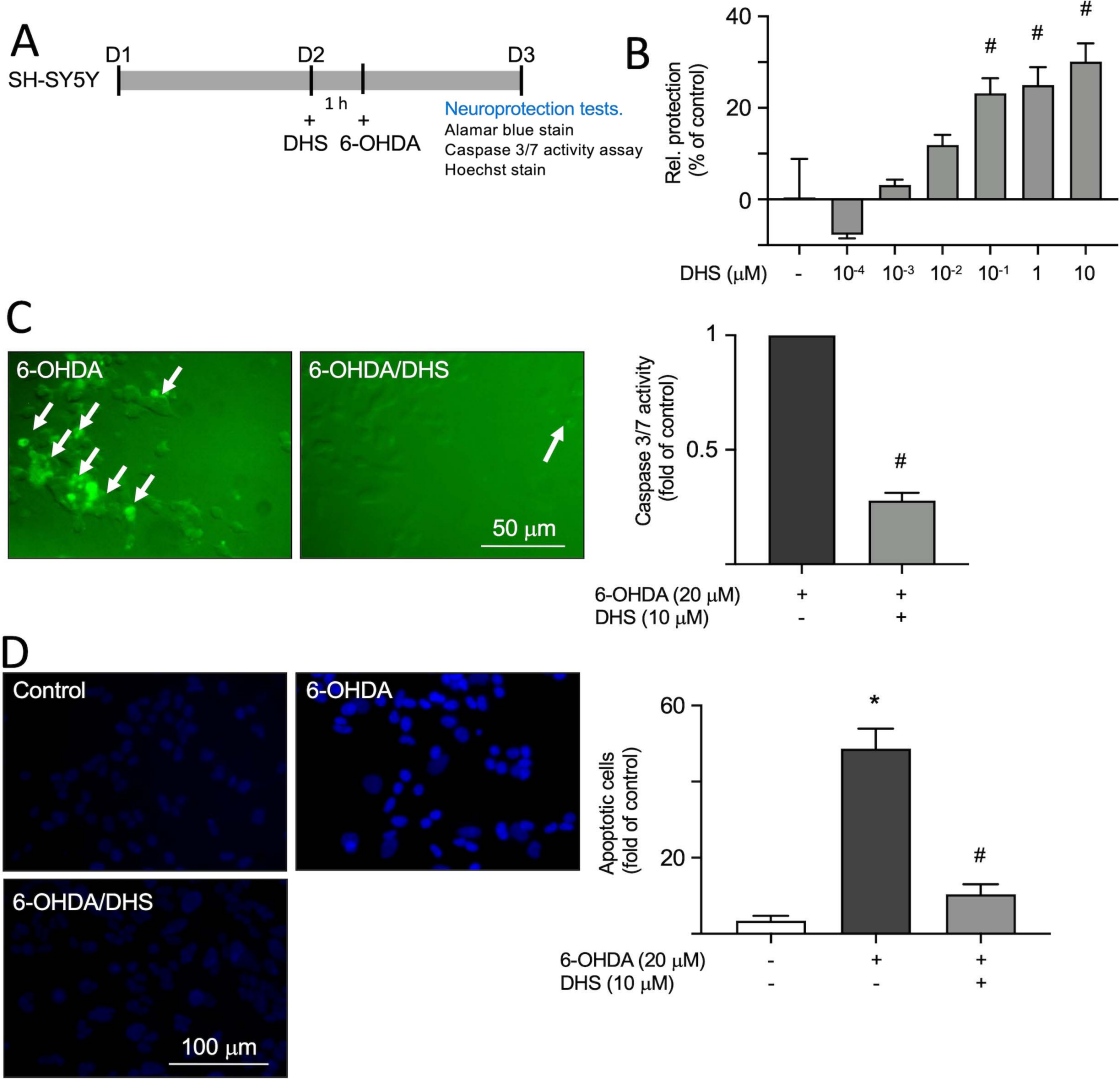
Neuroprotective effects of dihydrosinularin (DHS) in 6- hydroxydopamine (OHDA)- treated SH-SY5Y cells. (**A**) Experimental flow chart. On day 1, cells were plated. On day 2, after pretreatment with DHS for 1 h, neurotoxicity was induced with 6-OHDA for 16 h, and subsequently, cell viability (alamar blue stain), caspase 3/7 activity (CaspaTag™ Caspase 3, 7 *in situ* assay kit), and cell apoptosis (Hoechst stain) were assessed. (**B**) Assessment of cell viability with DHS (10^-4^-10 µM) treatment. The relative cell protection of untreated cells is presented as 0%. Rel protection: Relative protection; *p < 0.05 compared with the untreated group. (**C**) Microscopic images and quantitative analysis of the effects of DHS (10 µM) on caspase 3/7 activation in 6-OHDA-treated SH-SY5Y cells. Activated caspase 3/7 was visualized using fluorescence microscopy with a green fluorescence signal, indicated by white arrows. *p < 0.05 compared with the 6-OHDA alone group. (**D**) Microscopic images and quantitative analysis of DHS (10 µM) effect on cell apoptosis in 6-OHDA-treated SH-SY5Y cells. Hoechst staining (blue) was used to quantify the extent of cell apoptosis. *p < 0.05 compared with the untreated group; ^#^*p* < 0.05 compared with 6-OHDA alone group.

6-hydroxydopamine (6-OHDA)-treated SH-SY5Y cells are commonly utilized to induce neuronal death and establish an *in vitro* model of PD [9]. Thus, we used these cells as a drug screening platform (Fig. **2A**). Exposure to 20 µM of 6-OHDA resulted in the activation of apoptotic caspase 3 and caspase 7 in the SH-SY5Y cells, with a significant increase in their activation observed after 6, 8, and 12 h of treatment with 6-OHDA (from 1- to 6-7- fold, *p* = 0.007-0.040) using the CaspaTag™ Caspase 3, 7 *in Situ* Assay Kit analysis (Fig. **2B**). Following pretreatment with various concentrations (10^-4^-10 µM) of DHS for 1 h, 20 µM of 6-OHDA was utilized to evaluate the ability of DHS to prevent cell death induced by 6-OHDA (Fig. **3A**). When using 10^-4^-10 µM of DHS, the groups that received pretreatment showed a significant increase in relative cell viability rates compared with that in the untreated group (from 0 to 23-30%, *p*<0.001) (Fig. **3B**). Detecting the activation of caspase 3 and 7 within cells can be achieved with the CaspaTag™ Caspase 3, 7 *in situ* Assay Kit. The findings showed that when SH-SY5Y cells were pretreated with 10 µM of DHS for 1 h, the activation of caspase 3 and 7 was significantly inhibited (from 1- to 0.24-0.31- fold, *p*<0.001) (Fig. **3C**). Additionally, we used the Hoechst stain, a fluorescence dye capable of penetrating the cell membrane and binding to DNA, to determine the inhibition of cell apoptosis by DHS. Treatment with 20 µM of 6-OHDA induced nuclear structural changes associated with apoptosis, such as chromatin condensation, fragmentation, and the appearance of bright spots (from 3- to 48-fold, *p*<0.001). However, in groups pretreated with DHS at 10 µM, a significant decrease in the number of nuclei undergoing apoptosis was observed (from 48- to 10- fold, *p*<0.001) (Fig. **3D**). Thus, DHS effectively prevents apoptosis induced by 6-OHDA.

### 3.3 Enhancement of Cell Survival Signaling by DHS

**Fig. (4):**
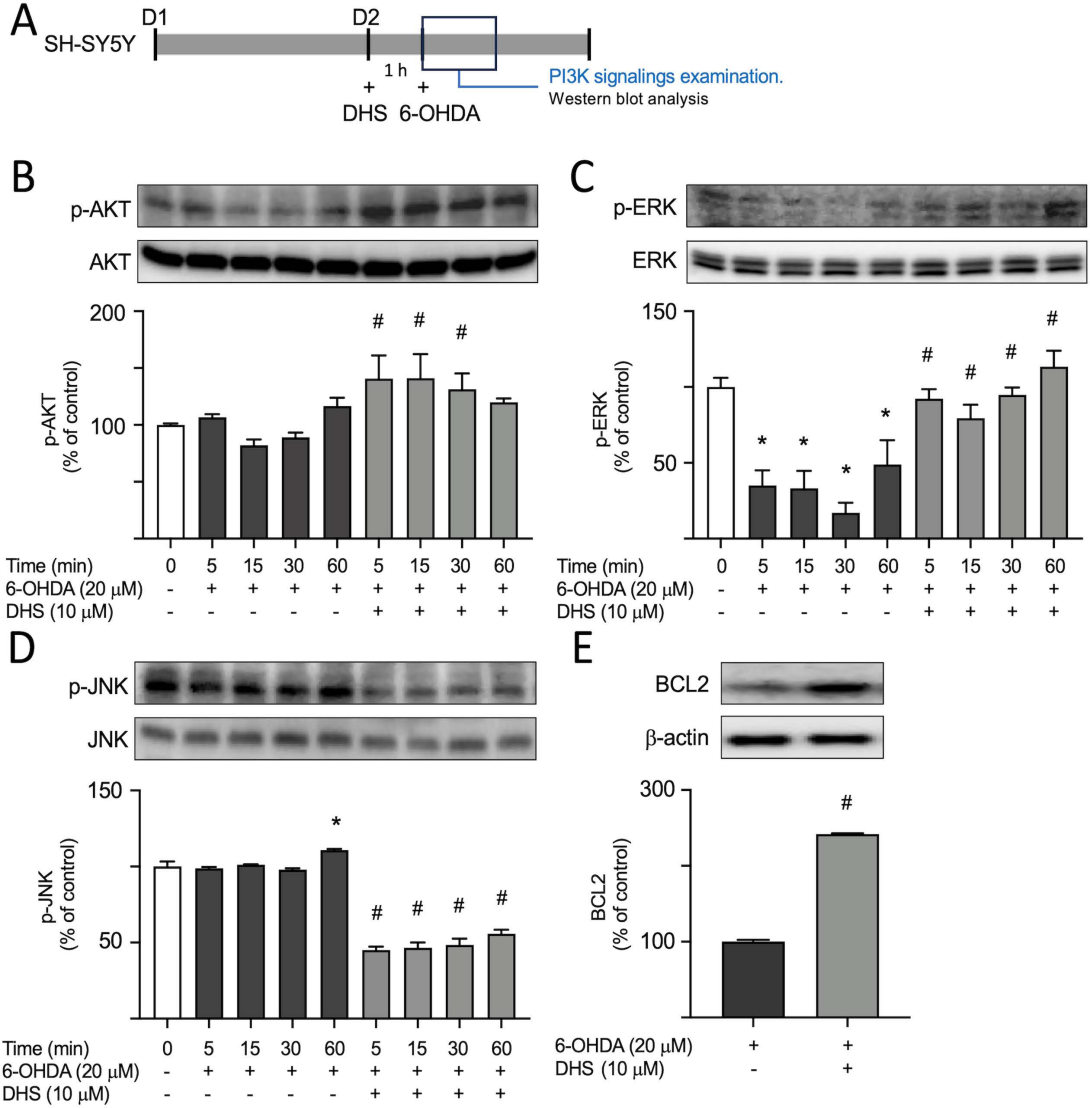
The effects of dihydrosinularin (DHS) on protein kinase B (AKT), extracellular signal-related kinase (ERK), c-Jun N-terminal kinase (JNK), and BCL2 signaling in 6- hydroxydopamine (OHDA)-treated SH-SY5Y cells. (**A**) Experimental flow chart. On day 1, cells were plated. On day 2, after pretreatment with DHS for 1 h, cells were subjected to 6- OHDA-induced neurotoxicity, and the levels of phosphorylated and non-phosphorylated AKT, ERK, and JNK were measured at 5, 15, 30, and 60 min. The BCL2 protein level was assessed at 6 h. (**B**) p-AKT (S473), AKT, (**C**) p-ERK (T202/Y204), ERK, (**D**) p-JNK (Thr183 and Tyr185), JNK, and (**E**) BCL2 levels analyzed by immunoblot using β-actin as a loading control. For normalization, protein expression levels in the untreated (**B**-**D**) or 6-OHDA alone group (**E**) cells are presented as 100%. (**B**-**D**), **Note:** *p < 0.05 compared with the untreated group; ^#^*p* < 0.05 compared with the 6-OHDA alone group at the same time point.

Western blot analysis was used to observe the phosphorylation levels of AKT and ERK in SH-SY5Y cells, as they play a crucial role in promoting cell survival through phosphorylation. As shown in (Fig. **4A**, **B**), in the groups exposed to 20 µM of 6-OHDA for 15 and 30 min, there was a decrease in p-AKT levels compared to the control group (from 100% to 82-89%, *p* = 0.605-0.953). However, when the DHS concentration was 10 µM, there was a significant enhancement in the expression level of p-AKT (from 82-106% to 131-141%, *p* = 0.034-0.036). Thus, DHS can significantly enhance AKT phosphorylation. The groups treated with 20 µM of 6-OHDA for 5-60 min exhibited a significant reduction in p-ERK levels compared to the control group (from 100% to 17-48%, *p*<0.001; Fig. **4C**). Conversely, treatment with DHS at 10 µM significantly elevated p-ERK expression (from 17-48% to 79-113%, *p*<0.001), indicating a substantial increase in ERK phosphorylation by DHS. Additionally, AKT phosphorylates and deactivates apoptosis signal-regulating kinase 1 (ASK1), which in turn inhibits JNK phosphorylation [31]. Groups treated with 20 µM of 6-OHDA for 60 min showed a significant increase in p-JNK levels (from 100 to 110%, *p* = 0.001; Fig. **4D**) compared to the control group. Alternatively, DHS administration at 10 µM significantly reduced p-JNK expression (from 97-110% to 44-55%, *p*<0.001), suggesting that it regulates JNK activity *via* the AKT pathway. As AKT is known to upregulate the expression of BCL2, subsequent analysis of the protein levels of BCL2 was conducted. Treatment with 10 μM of DHS significantly attenuated the decrease in BCL2 expression induced by 6-OHDA (from 100% to 241%, *p* = 0.034-0.036; Fig. **4E**), indicating its potential influence on BCL2 expression through the AKT pathway.

### 3.4 DHS Reduced 6-OHDA-induced Nuclear Translocation of The Nuclear Factor Kappa-light-chain- enhancer of Activated B Cells (NF-κB)

Previous studies have suggested a link between neuronal apoptosis induced by 6-OHDA and the excessive activation of NF-κB [28]. Additionally, JNK can activate NF-κB signaling, leading to increased nuclear translocation [32]. Therefore, we investigated the effect of DHS on NF-κB nuclear translocation. As shown in Fig. (**5**), using immunofluorescence assays, we showed that treatment with 20 µM of 6-OHDA alone led to a significant increase in the fraction of NF-κB nuclear translocation (from 23% to 48%, *p* = 0.006). Pretreatment with 10 µM of DHS effectively attenuated the nuclear translocation of NF-κB induced by 6-OHDA (from 48% to 27%, *p* = 0.015).

### 3.5 Inhibition of PI3K Attenuates DHS-induced Neuroprotection

**Fig. (6):**
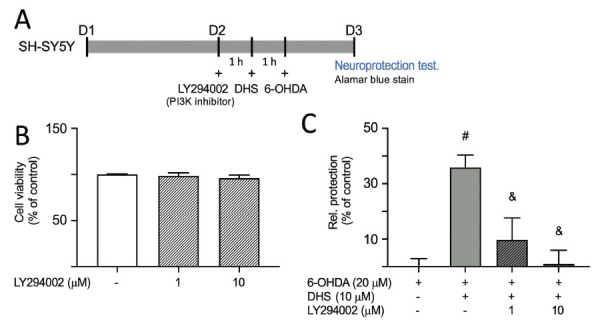
The effects of PI3K inhibition on neuroprotective effects of dihydrosinularin (DHS) in 6- hydroxydopamine (OHDA)-treated SH-SY5Y cells. (**A**) Experimental flow chart. On day 1, cells were plated. On day 2, cells were pre-treated with LY294002 (PI3K inhibitor) for 1 h, followed by DHS treatment for 1 h, then induced with 6-OHDA for 16 h to induce neurotoxicity, and cell viability was assessed using alamar blue staining. (**B**) LY294002 cytotoxicity against SH-SY5Y cells using alamar blue assay. Cells treated with LY294002 (1 and 10 µM) for 18 h. For normalization, the relative viability of untreated cells is presented as 100%. (**C**) Assessment of cell viability with LY294002 (1 and 10 µM) and DHS (10 µM) treatment. The relative cell protection of untreated cells is normalized at 0%. ^#^*p* < 0.05 compared with the 6-OHDA alone group; ^&^*p* < 0.05 compared with the 6-OHDA+DHS group.

**Fig. (7):**
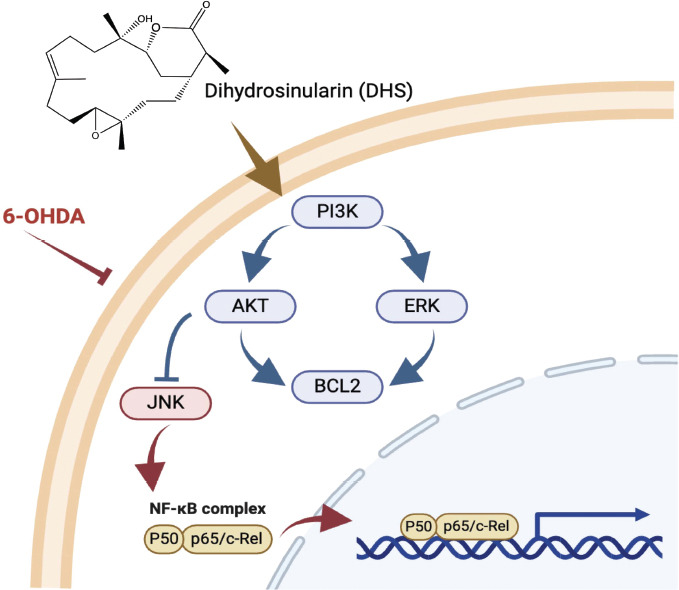
Neuroprotective mechanisms of coral-derived dihydrosinularin (DHS) in 6- hydroxydopamine (OHDA)-induced neurotoxicity in SH-SY5Y cells. DHS activates the PI3K signaling pathway, which results in the restoration of protein kinase B (AKT) and extracellular signal-related kinase (ERK) levels reduced by 6-OHDA, along with a decrease in the heightened c-Jun N-terminal kinase (JNK) expression induced by 6-OHDA. Consequently, it enhances the expression of BCL2, ultimately protecting neuronal cells by inhibiting caspase activity and preventing cell apoptosis.

Given the neuroprotective effects of DHS against 6-OHDA-induced neurotoxicity, we inhibited PI3K function using its inhibitor, LY294002, to assess the potential of PI3K and downstream signaling as therapeutic targets of DHS (Fig. **6A**). First, we assessed the toxicity of LY294002 on SH-SY5Y cells. LY294002 at 1 µM and 10 µM did not have any detrimental effect on cell viability (Fig. **6B**). Pretreatment with DHS at 10 µM effectively prevented cell death induced by 6-OHDA (35%, *p*<0.001) (Fig. **6C**). However, this neuroprotective effect was reduced by the administration of LY294002 at 1 µM and 10 µM (1-9%, *p*<0.001). Thus, the activation of PI3K is important in the signaling pathway utilized by DHS to protect neurons, indicating that DHS enhances cell survival by activating the PI3K signaling pathway (Fig. **7**).

## 4 DISCUSSION

This study primarily utilized cell viability analysis, western blotting, and immunofluorescence staining to explore neuroprotective signaling pathways, highlighting the neuroprotective capability of DHS. In the screening of various marine natural compounds, DHS stood out for its favorable bioavailability and BBB permeability, as revealed by physicochemical properties analysis. DHS activates the PI3K signaling pathway, resulting in increased expression of phosphorylated AKT and ERK, decreased expression of phosphorylated JNK, and subsequent enhancement of BCL2 expression, ultimately protecting neuronal cells by inhibiting caspase and preventing cell apoptosis. These findings reveal its ability to enhance pro-survival pathways while suppressing neuronal pro-apoptotic signaling pathways.

6-OHDA, a neurotoxin, selectively damages dopaminergic neurons and induces apoptosis in both cellular and animal models [33]. This characteristic has rendered it a valuable tool for evaluating potential treatments for PD. Moreover, 6-OHDA enters neuronal cells and disrupts ATP production by inhibiting the mitochondrial respiratory chain complex I, resulting in the production of reactive oxygen species (ROS) [34]. ROS induces a transient opening of the mitochondrial permeability transitory pore, altering mitochondrial membrane permeability and facilitating the release of factors like cytochrome c into the cytoplasm, activating caspase 3 and ultimately leading to cell death [35, 36]. In this study, it was observed that the treatment of human neuroblastoma cells (SH-SY5Y) with 20 µM of 6-OHDA resulted in a significant decrease in cell viability and an evident rise in caspase 3 activity. This finding is consistent with prior research that has observed the activation of cell apoptosis by 6-OHDA [27, 36, 37]. Despite this, pretreatment with DHS for 1 h effectively prevented the decrease in cell viability, activation of caspase 3, and the resulting apoptosis caused by 20 µM of 6-OHDA.

By conducting further experiments, we aimed to understand how DHS can resist neuronal apoptosis caused by 6-OHDA. Our focus was on modulating the signaling pathways associated with cell apoptosis and uncovering the molecular mechanisms that contribute to the cellular protection provided by DHS. Previous research has shown that activating the PI3K/AKT signaling pathway can be a promising strategy for treating neuronal apoptosis caused by 6-OHDA [12, 38]. When PI3K is inhibited, it leads to a decrease in ERK activation, which in turn has an impact on the plasticity of neurons [39]. Moreover, AKT triggers JNK activation, which subsequently induces various stress responses, including cell death, differentiation, and the release of inflammatory cytokines [31]. Following activation, JNK inhibits the generation of BCL2 and BCL-xL proteins, consequently enhancing the activation of BAD and BAX, ultimately resulting in cell apoptosis [15, 40]. In the present study, pretreatment with DHS effectively restored the decreased phosphorylation of AKT and ERK, along with the expression of BCL2 induced by 6-OHDA.

Furthermore, it inhibited the activation of caspase 3 and caspase 7. Previous research has demonstrated that the use of selective and reversible inhibitors targeting caspase 3 and caspase 7 can play a significant role in neuroprotection, as well as in the delay of cell death and brain damage [41]. We conducted experiments using the PI3K inhibitor LY294002 to explore the potential neuroprotective effects of DHS through the regulation of the PI3K signaling pathway. The findings indicated a notable inhibition in the ability of DHS to improve the neurotoxicity induced by 6-OHDA. Based on these results, it appeared that DHS had the ability to influence the activation of AKT, ERK, and JNK *via* the PI3K signaling pathway, which subsequently enhanced the anti-apoptotic properties of BCL2, ultimately leading to neuroprotection.

In *in vitro* models of PD, the induction of neuronal apoptosis by 6-OHDA and the subsequent activation of NF-κB highlight the importance of NF-κB activation in this process [28, 42]. Research has revealed that NF-κB activation can result in its movement to the nucleus and interaction with certain genes, which in turn stimulates the production of molecules that prevent cell death and support cell survival [43, 44]. Moreover, research has indicated that NF-κB can be triggered by neurotoxins like glutamate, leading to the expedited formation of the NF-κB family protein complex, which consists of p50 and p65, to promote cell apoptosis [45]. In such cases, inhibiting NF-κB activation has been found to protect neurons from glutamate-induced damage [42, 46]. This study provided evidence that 6-OHDA causes an elevation in NF-κB nuclear translocation; however, DHS administration mitigates this increase, exerting a protective effect on neurons.

Despite the plethora of marine compounds, very few have been investigated for clinical applications in neurodegenerative diseases [19]. For example, leucettinib-21 derived from sponges and bryostatin derived from bryozoans are currently under investigation in clinical phase I trials as potential treatments for AD [47, 48]. The marine compounds currently being investigated in preclinical studies for PD treatment are astaxanthin and SV2-1. Astaxanthin, found in various aquatic organisms, exhibits neuroprotective properties by inhibiting apoptosis, mitochondrial abnormalities, and intracellular ROS formation in neuroblastoma SH-SY5Y cells treated with 6-OHDA [49]. SV2-1, a heteropolysaccharide derived from the squid *Ommastrephes bartrami*, demonstrated protective effects against 6-OHDA-induced cell death in pheochromocytoma PC12 cells, concurrently enhancing superoxide dismutase activity and reducing malondialdehyde levels [50]. Based on Lipinski's rule and tPSA, DHS showed better bioavailability and blood-brain barrier permeability compared to marine compounds like astaxanthin and SV2-1 currently in preclinical research for PD treatment [49, 50], therefore, DHS holds great promise. Future work will be warranted to determine the effects of DHS on PD animal models.

In a previous study, five compounds with a similar chemical structure to DHS were identified [22]. These compounds were extracted from the soft coral of the same genus. The study reveals that minor differences in their structures can lead to variations in biological activities, such as anti-inflammatory and cell proliferation properties [22]. In our preliminary anti-inflammatory screening, sinularin, 11-episinulariolide acetate, 11-dehydrosinulariolide (11-de), and DHS have demonstrated the ability to inhibit LPS-induced proinflammatory iNOS protein expression in macrophages. Notably, sinularin and 11-episinulariolide acetate were previously found to have relief effects for analgesic [51, 52] and rheumatoid arthritis [53], respectively, but without neuroprotective properties. On the other hand, 11-de [53] and DHS were found to have neuroprotective properties. In the future, we aim to work with chemical scientists to gather a broader range of membrane-type compounds resembling DHS and investigate their potential for neuroprotective activity. Our objective is to identify the key chemical structures of these compounds. Additionally, 11-de showed promising anti-apoptotic and anti-inflammatory properties and was found to enhance motor function in zebrafish and rats treated with 6-OHDA [11, 54, 55]. Both 11-de and DHS meet Lipinski's rule and exhibit the potential for crossing the BBB. They both exert neuroprotective effects through PI3K-related pathways; however, only DHS is known to regulate JNK phosphorylation. Studies have indicated the IC_50_ of 11-de in small-cell lung cancer (29-43 μM), melanoma (17.4 μM), leukemia (6.9-12.2 μM), and oral cancer (10 μM) cell lines [53, 56-58]. 11-de demonstrated higher cytotoxicity than DHS (IC_50_ >200μM), suggesting that DHS holds greater potential for neuroprotective drug development.

There are three potential paths ahead. First, further investigation is required to understand the exact therapeutic targets through which DHS provides neuroprotective effects. Further investigation may involve conducting in-depth examinations of downstream signaling pathways, such as PI3K/AKT, ERK, JNK, and BCL2, to gain a deeper understanding of how these pathways contribute to the neuroprotective effects of DHS. Second, DHS derivatives or analogs with enhanced potency and specificity for neuroprotection should be developed. One possible approach is to utilize medicinal chemistry approaches to enhance the medicinal properties of DHS, making it more suitable for clinical applications. Third, the potential synergistic effects of DHS with existing PD medications or other neuroprotective agents should be investigated. Combination therapies have the potential to provide greater effectiveness and fewer side effects in patients with PD. Overall, in the realm of neuroscience, delving deeper into DHS and understanding how it works could pave the way for novel strategies for treating PD.

## CONCLUSION

The marine natural compound DHS demonstrated the ability to protect neurons by mitigating the damage caused by 6-OHDA in SH-SY5Y neuroblastoma cells. Through the activation of the PI3K signaling pathway, this protective mechanism worked to inhibit neuronal apoptosis, thus protecting neuronal cells. DHS has shown remarkable neuroprotective properties, which could make it an attractive option for treating PD.

## Figures and Tables

**Fig. (5):**
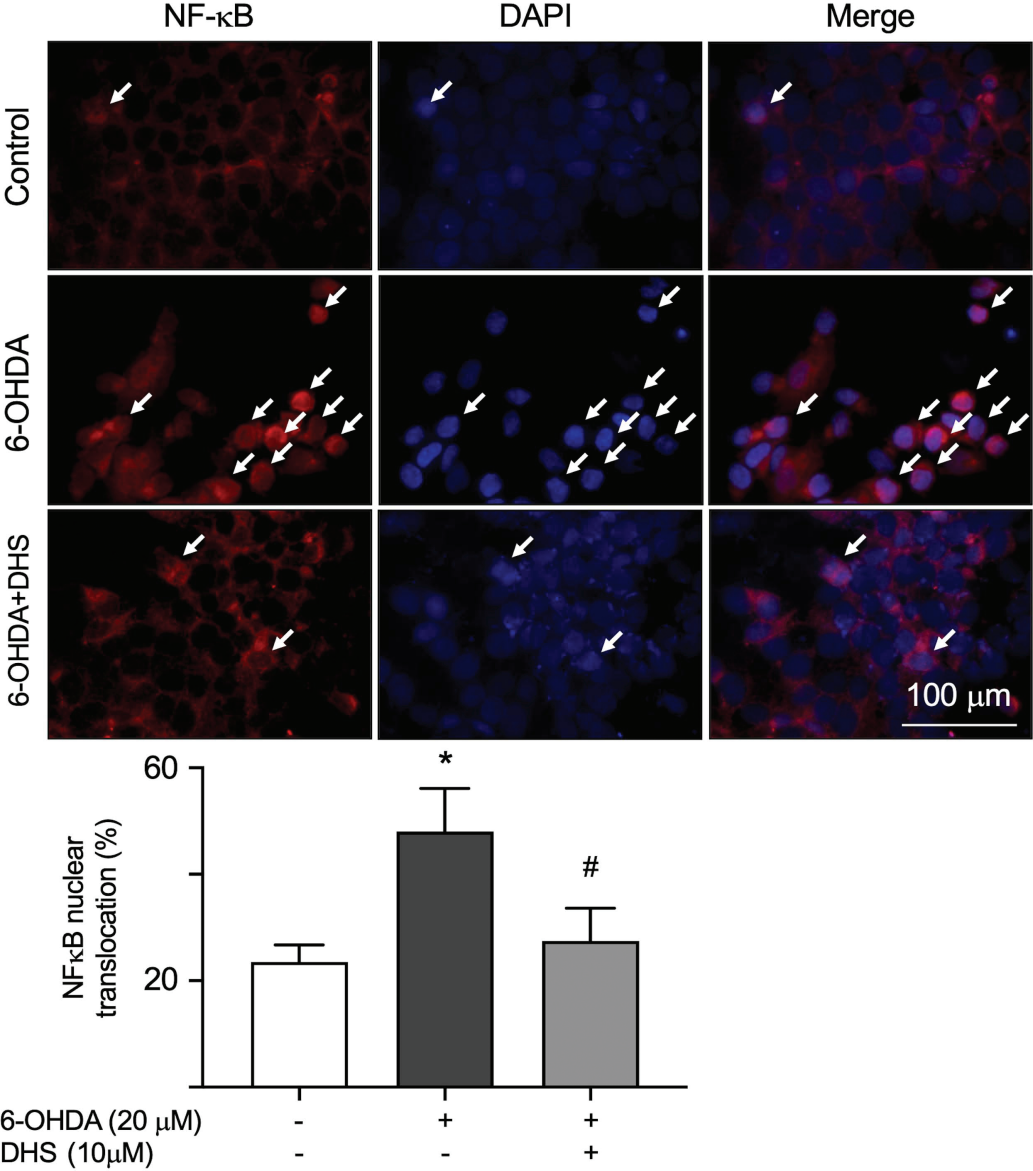
The effect of dihydrosinularin (DHS) on nuclear factor kappa beta (NF-κB) nuclear translocation in 6- hydroxydopamine (OHDA)-treated SH-SY5Y cells. NF-κB staining (red was used to quantify the extent of its nuclear translocation. Nuclei were counterstained with 4′,6-diamidino-2-phenylindole (DAPI) (blue). **Note:** *p < 0.05 compared with the untreated group; ^#^*p* < 0.05 compared with the 6-OHDA alone group.

## Data Availability

The data and supportive information are available within the article.

## LIST OF ABBREVIATIONS

PD: Parkinson's Disease
AD: Alzheimer's Disease
6-OHDA: 6-Hydroxydopamine
ERK: Extracellular Signal-related Kinase
DHS: Dihydrosinularin
MW: Molecular Weight
FBS: Fetal Bovine Serum
