# Supplementary material for: Soft Corals-derived Dihydrosinularin Attenuates Neuronal Apoptosis in 6-hydroxydopamine-induced Cell Model of Parkinson's Disease by Regulating the PI3K Pathway
Source: Curr Med Chem. 2024 Sep 3;32(8):1606–20. doi: 10.2174/0109298673323198240823070219 (PMC12246742; doi:10.2174/0109298673323198240823070219)
Supplement: Supplementary file 1 [file CMC-32-8-1606_SD1.pdf]

Supplementary Material

Soft Corals-derived Dihydrosinularin Attenuates Neuronal Apoptosis in 6-hydroxydopamine-induced Cell Model of Parkinson's Disease by Regulating the PI3K Pathway

Zhi-Hong Wen[1,#], Ya-Jen Chiu[2,#], San-Nan Yang[3], Tzu-Yi Huang[1], Chien-Wei Feng[4], Nan-Fu Chen[5], Chun-Sung Sung[6] and Wu-Fu Chen[1,2,*]

[1]Department of Marine Biotechnology and Resources, National Sun Yat-Sen University, Kaohsiung, 804201, Taiwan; [2]Department of Neurosurgery, Kaohsiung Chang Gung Memorial Hospital and Chang Gung University College of Medicine, Kaohsiung, 833301, Taiwan; [3]Department of Pediatrics, E-DA Hospital, School of Medicine, College of Medicine I-Shou University, Kaohsiung, 82445, Taiwan; [4]Department of Obstetrics and Gynecology, Kaohsiung Medical University Hospital, Kaohsiung Medical University, Kaohsiung, 80756, Taiwan; [5]Department of Surgery, Division of Neurosurgery, Kaohsiung Armed Forces General Hospital, Kaohsiung, 80284, Taiwan; [6]Department of Anesthesiology, Division of Pain Management, Taipei Veterans General Hospital, Taipei, 112201, Taiwan

(A)

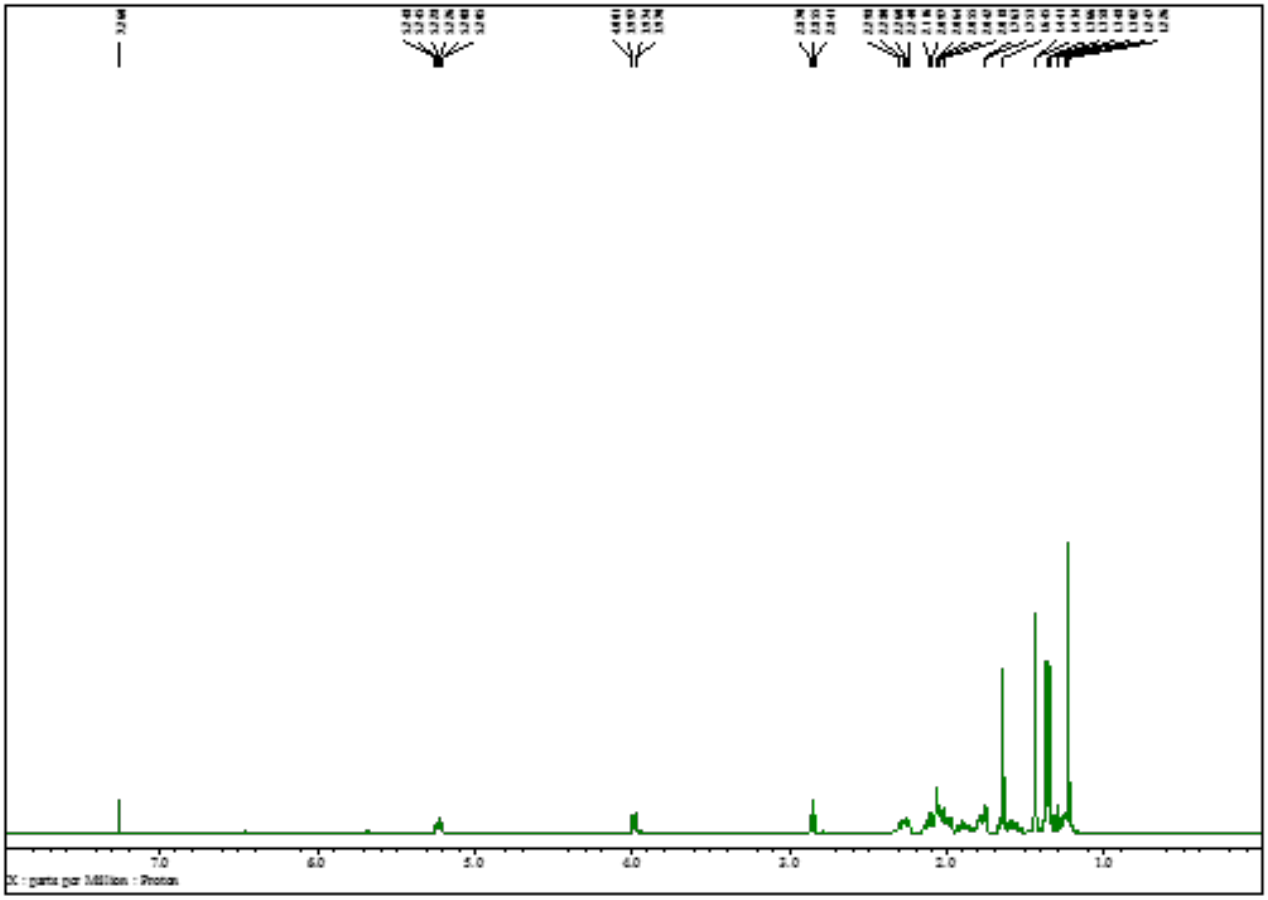

(B)

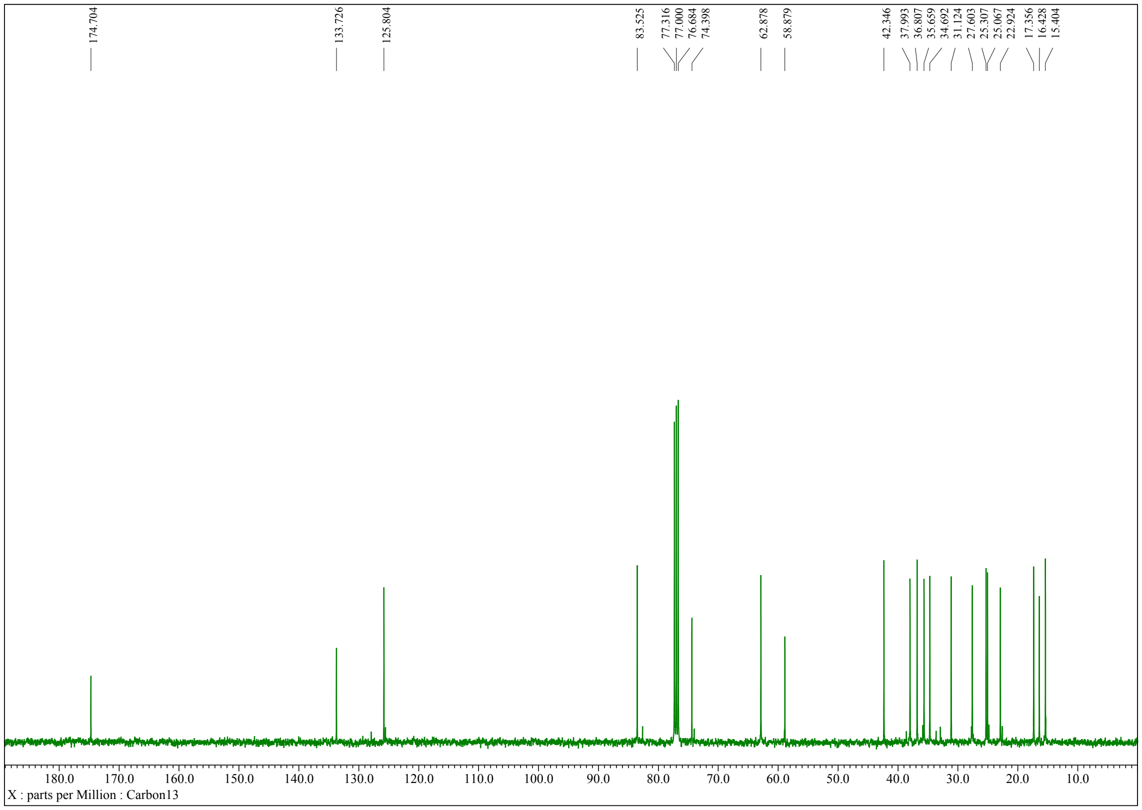

(C)

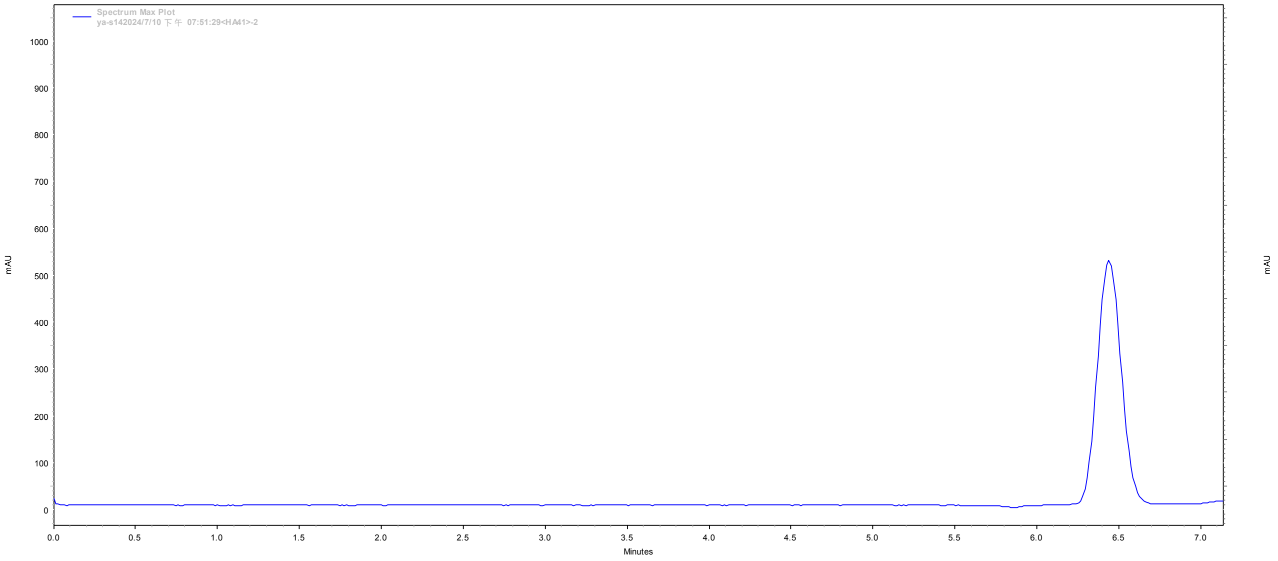

Fig. (S1). H-NMR, C-NMR, and HPLC spectrum of dihydrosinularin (DHS). (**A**) ^{1}H NMR spectrum (400 MHz) of DHS in $CDCl_3$. (**B**) ^{13}C NMR spectrum (100 MHz) of DHS in $CDCl_3$. (**C**) The purity of DHS was identified by NP-HPLC using n-hexane: acetone (4:1, flow rate: 3 mL/min).
